# Chronic upper airway inflammation related to high Th2 cytokines in Mendelian susceptibility to mycobacterial disease case

**DOI:** 10.5339/qmj.2022.fqac.24

**Published:** 2022-04-04

**Authors:** Ibtihal Benhsaien, Rui Yang, Fatima Ailal, Marc Weisshaar, Federico Mele, Jean-Laurent Casanova, Jacinta Bustamante, Ahmed Bousfiha

**Affiliations:** ^1^Laboratory of Clinical Immunology, Inflammation, and Allergy, Faculty of Medicine and Pharmacy of Casablanca, King Hassan II University, 20460 Casablanca, Morocco E-mail: ibtihalbenhsaien@gmail.com; ^2^Clinical Immunology Unit, Department of Pediatric Infectious Diseases, Children’s Hospital, CHU Averroes, 20460 Casablanca, Morocco; ^3^St Giles Laboratory of Human Genetics of Infectious Diseases, Rockefeller Branch, Rockefeller University, New York, NY 10065, USA; ^4^Institute of Microbiology, ETH Zurich, CH-8093 Zurich, Switzerland; ^5^Center of Medical Immunology, Institute for Research in Biomedicine, Faculty of Biomedical Sciences, University of Italian Switzerland (USI), CH-6500 Bellinzona, Switzerland

**Keywords:** MSMD, Th2 cytokines, UAI

## Abstract

In this report, we have described a child suffering from Mendelian susceptibility to mycobacterial disease (MSMD) owing to an autosomal recessive, complete T-bet deficiency, which impairs IFN-γ production by innate and innate-like adaptive, but not mycobacterial-reactive purely adaptive lymphocytes. In this study, we explored the persistent upper airway inflammation (UAI) and blood eosinophilia in this patient. Unlike the wild-type (WT) T-bet, the mutant form of T-bet from this patient did not inhibit the production of T helper 2 (Th2) cytokines, including IL-4, IL-5, IL-9, and IL-13, when overexpressed in Th2 cells. Moreover, *Herpesvirus saimiri* immortalized T cells from the patient produced abnormally large amounts of Th2 cytokines, and the patient had markedly high plasma IL-5 and IL-13 concentrations. Finally, the patient’s CD4^+^ αβ T cells produced most of the Th2 cytokines in response to chronic stimulation, regardless of their antigen specificities, a phenotype reversed by the expression of WT T-bet. T-bet deficiency thus underlies the excessive production of Th2 cytokines, particularly IL-5 and IL-13, by CD4^+^ αβ T cells, causing blood eosinophilia and UAI. The MSMD of this patient results from defective IFN-γ production by innate and innate-like adaptive lymphocytes, whereas the UAI and eosinophilia result from excessive Th2 cytokine production by adaptive CD4^+^ αβ T lymphocytes.

